# Fragile X Syndrome

**Published:** 2014-12-30

**Authors:** Wilmar Saldarriaga, Flora Tassone, Laura Yuriko González-Teshima, Jose Vicente Forero-Forero, Sebastián Ayala-Zapata, Randi Hagerman

**Affiliations:** 1 Professor Morphology, Gynecology and Obstetrics Hospital Universitario del Valle, Universidad del Valle, Cali, Colombia.; 2 Department of Biochemistry and Molecular Medicine; UC Davis MIND Institute, University of California, Sacramento, USA.; 3 Medicine and surgery School, University of Valle. Cali, Colombia.; 4 Medical director and distinguished professor. Holder Research Chair in Fragile X syndrome. Department of Pediatrics, School of Medicine. UC Davis MIND Institute, University of California, Davis, United States.

**Keywords:** Fragile X Syndrome, Fragile X Mental Retardation Protein, intellectual disability, Therapeutics, Genetic Counselling

## Abstract

Fragile X Syndrome (FXS) is a genetic disease due to a CGG trinucleotide expansion, named full mutation (greater than 200 CGG repeats), in the fragile X mental retardation 1 gene locus Xq27.3; which leads to an hypermethylated region in the gene promoter therefore silencing it and lowering the expression levels of the fragile X mental retardation 1, a protein involved in synaptic plasticity and maturation. Individuals with FXS present with intellectual disability, autism, hyperactivity, long face, large or prominent ears and macroorchidism at puberty and thereafter. Most of the young children with FXS will present with language delay, sensory hyper arousal and anxiety. Girls are less affected than boys, only 25% have intellectual disability. Given the genomic features of the syndrome, there are patients with a number of triplet repeats between 55 and 200, known as premutation carriers. Most carriers have a normal IQ but some have developmental problems. The diagnosis of FXS has evolved from karyotype with special culture medium, to molecular techniques that are more sensitive and specific including PCR and Southern Blot. During the last decade, the advances in the knowledge of FXS, has led to the development of investigations on pharmaceutical management or targeted treatments for FXS. Minocycline and sertraline have shown efficacy in children.

## Abstract (es)

El Síndrome de X Frágil (SXF), es una enfermedad genética debida a una expansión del trinucleótido CGG, nombrada mutación completa (más de 200 repeticiones de CGG) en el locus Xq27.3 del gen X fragil de retardo mental; la cual lleva a una hipermetilación de la región promotora del gen, silenciándolo y disminuyendo los niveles de expresión de la proteína X frágil de retardo mental 1, relacionada con la plasticidad y maduración neuronal. Los individuos con SXF presentan retardo mental, autismo, hiperactividad, cara alargada, orejas grandes o prominentes y macroorquidismo desde la pubertad. La mayoría de niños con SXF presentan retraso en el lenguaje, hiperactivación sensorial y ansiedad. Las niñas se afectan menos que los varones, solo el 25% presenta retardo mental. Dadas las características genómicas del síndrome, existen pacientes con un número de repetición de la tripleta entre 55 y 200 que se denominan portadores de la premutación. La mayoría de los portadores tienen un coeficiente intelectual normal, pero presentan problemas en el desarrollo. El diagnóstico en SXF ha evolucionado del cariotipo con medio especial de cultivo, a pruebas moleculares más sensibles y específicas incluyendo PCR y Southern blot. Durante la última década, los avances en el conocimiento sobre el SXF han permitido el desarrollo de investigaciones sobre el manejo farmacológico o tratamientos específicos para el SXF. La minociclina y la sertralina han demostrado eficacia en niños.

## Introduction 

The Fragile X Syndrome (FXS) is a genetic disease inherited through the X chromosome, which was described for the first time in 1943 by Martin and Bell 1. It is actually considered the most common inherited cause of intellectual disability and the second most prevalent cause after Down syndrome. Most cases of Down syndrome are de novo but FXS is always inherited with many individuals in the family tree, either affected or a carrier of FXS. Affected men have a classic phenotype characterized by long face, large and protruding ears and macroorchidism 2. The Fragile X Syndrome is caused by an abnormal expansion in the number of the trinucleotide CGG repeats located in the 5' UTR in the fragile X mental retardation 1 gene (*FMR1*) at Xq27.3. It is a dynamic mutation with expansion of the CGG repeat in each generation moving from the premutation range of 55 to 200 repeats and expanding to a full mutation when pass on by a women to her children 3.

Patients affected with FXS have more than 200 repeats of the CGG trinucleotide. On the other hand, premutation carriers (55 to 200 repeats) although are not affected with the classic FXS phenotype, can have other medical, psychiatric and neurological problems. In the last 15 years multiple advances have been made in the description of genetic characteristics, function of the protein encoded by the *FMR1 *gene (FMRP), pharmacological management and the description, in carriers of the premutation, of the Fragile X associated Tremor/Ataxia Syndrome (FXTAS) and fragile X-associated primary ovarian insufficiency (FXPOI) 3 ^-^ 5.

The objective of this review is to contribute to the dissemination of knowledge on FXS among health professionals and thus improving the diagnosis and management of these patients. 

## 1. Epidemiology

The actual worldwide prevalence, determined by molecular assays, it's estimated in one per 5,000 men 6 and in one per 4,000 to 6,000 women 6 ^,^ 7. Ricaurte is a district of the municipality of Bolívar, located in the north of Valle del Cauca, in which there has been identified a high prevalence of mental disability, 39 intellectually disable individuals in 1124 habitants 8. During the late 1990's, a study found that the cause of this disability in the region was FXS. In this study 19 patients were diagnosed with the syndrome by karyotype with G bands in folate deficient medium; furthermore clinical diagnosis was done in 16 more patients in whom the karyotype was not performed. The cases were found in 3 family names and a possible common ancestor was postulated given the migratory patterns and founding characteristics of the town 9. By the year 1999 the prevalence of FXS in Ricaurte was determined as 1:38 men and 1:100 women, which exceeded 100 times the prevalence reported in literature.

A number of medical conditions and syndromes, associated with carriers of the premutation including depression, anxiety, migraine headaches, hypertension, sleep apnea, immune mediated diseases including hypothyroidism and fibromyalgia, and FXTAS and FXPOI have been described in the past 10 years 10. The prevalence of the premutation in the general population is 1:130-200 women and 1:250 to 450 men 11 ^,^ 12. Tremor/Ataxia Syndrome occurs in approximately 40% of men with the premutation and 16% of women, whereas FXPOI occurs in approximately 16 to 20% of women with the premutation 2 ^,^ 10.

## 2. Genomics

 The Fragile X Syndrome is caused by an alteration in the *FMR1* gene, with locus Xq27.3. This gene harbors a CGG repeat within the 5' Untranslated Region. Depending on the number of repetitions, 4 types of alleles are defined with different clinical manifestations 3 ^,^ 13: Normal alleles, up to 44 CGG repeats; premutation (PM) alleles, between 55 and 200 and full mutation alleles (FM) with more than 200 repeats. The fourth type of allele is named "grey zone" or intermediate allele and contains between 45 and 54 repeats and it has been proposed as a precursor for PM alleles. 

The silencing of the *FMR1 *gene is the result of a series of complex epigenetic modifications following the expansion of the trinucleotide repeat 14. The FM alleles undergo a methylation process in the CpG island within the gene promoter and in the CGG repeats 15 ^,^ 16. In male patients with the FM allele, every cytosine within the CpG island is methylated, contrarily to healthy individuals who lack of any methylation 17. A recently discovered boundary sequence in the *FMR1 *gene found 650 to 800 nucleotides upstream from the repeated region that suffers methylation. This boundary sequence, because of its chromatin interaction, limits the hypermethylated region of the genome, protecting the promoter of *FMR1* from possible methylation 15 ^,^ 16 ^,^ 18. In individuals with FXS the methylated boundary sequence is lost allowing the expansion of the methylation up to the promoter of the *FMR1* gene. This findings strongly suggest that changes in the sequence of nucleotides and conformational structure of the chromatin of the boundary sequences would favor the epigenetic changes that would induce *FMR1 *silencing and ultimately preventing FMRP production 19.

 The Fragile X Syndrome is usually caused by the methylation and gene silencing associated with the full mutation although deletions of the cod ing region of the gene can also lead to absence of FMRP. In addition, point mutations or reading frame shifts can also occur leading to a functional deficit of the protein FMRP and the consequent phenotype; however, these genomic changes account only for less than 1% of all FXS cases described 20.

The clinical involvement in those with a premutation is caused by a different mechanism than FXS, which is elevated *FMR1* mRNA levels leading to RNA toxicity. 2-8 folds normal levels of mRNA are observed in premutation carriers and the excess such mRNA lead to the sequestration of important proteins for neuronal function by the hairpin structures that form in the CGG repeats. RNA toxicity causes the neurons to die earlier in culture and so the carriers are at risk for late onset disorders including FXTAS and FXPOI 10.

The FMRP is a RNA binding protein that shows preference towards RNA homopolymers 21 as for certain subgroups of cerebral transcripts 22. The protein encoded by the FMR1 gene is involved in the regulation of the RNA stability, subcellular transport and translation of neural mRNAs that codify for proteins involved in the synapsis development, neural plasticity and brain development 23 ^-^ 25. Various studies have revealed that in the absence of this protein, a wide range of neural mRNAs are altered, augmenting neural protein synthesis and resulting in dendritic spine dysmorphogenesis and an excitation/inhibition imbalance (Glutamate/GABA), phenomena present in FXS 26 ^,^ 27. The dendritic spine dysmorphogenesis plays a role in the clinical manifestations of the syndrome, due to the weak synaptic connections leading to intellectual deficits and behavioral problems. Multiple neurotransmitter systems are impaired because of the lack of FMRP and there is enhanced protein production in the hippocampus and throughout the brain 3. 

## 3. Heritability

FXS heritability does not have a Mendelian classic inheritance pattern. It depends on the number of trinucleotide CGG repeats within the promoter of the *FMR1* gene 28. The transition from PM to FM alleles occurs because of expansion phenomena during the transmission of the maternal X chromosome carrying PM, to her children 2. This expansion does not occur during the transmission of the paternal X chromosome, with the PM, to their daughters 28. So all daughters of men with the premutation will be obligate carriers of the premutation but then these daughters have a 50% risk to have children with FXS. 

### 3.1. Dynamic of the mutation

The risk of transition from PM to FM in the descendants depends on the number of trinucleotide repeats in the PM allele, reaching ~100% for PM alleles with more than 99 repeats 29 ^,^ 30. The expansion from PM to FM in meiosis can occur in alleles with as little as 56 CGG repeats 31; the odds that this occurs depends on the range of repeats in which the patient is classified, number of AGG interruptions and age of the mother (Table 1) 29 ^,^ 30. Normally there is an AGG anchor with every 9 or 10 CGG repeats in *FMR1*. The anchors can modify the risk for expansion of the CGG repeat when passed on by the mother. Women with the premutation and 2 AGG anchors have a lower risk of expansion to the full mutation compared to women with no AGG anchors 29 ^,^ 30.

**Table 1.**
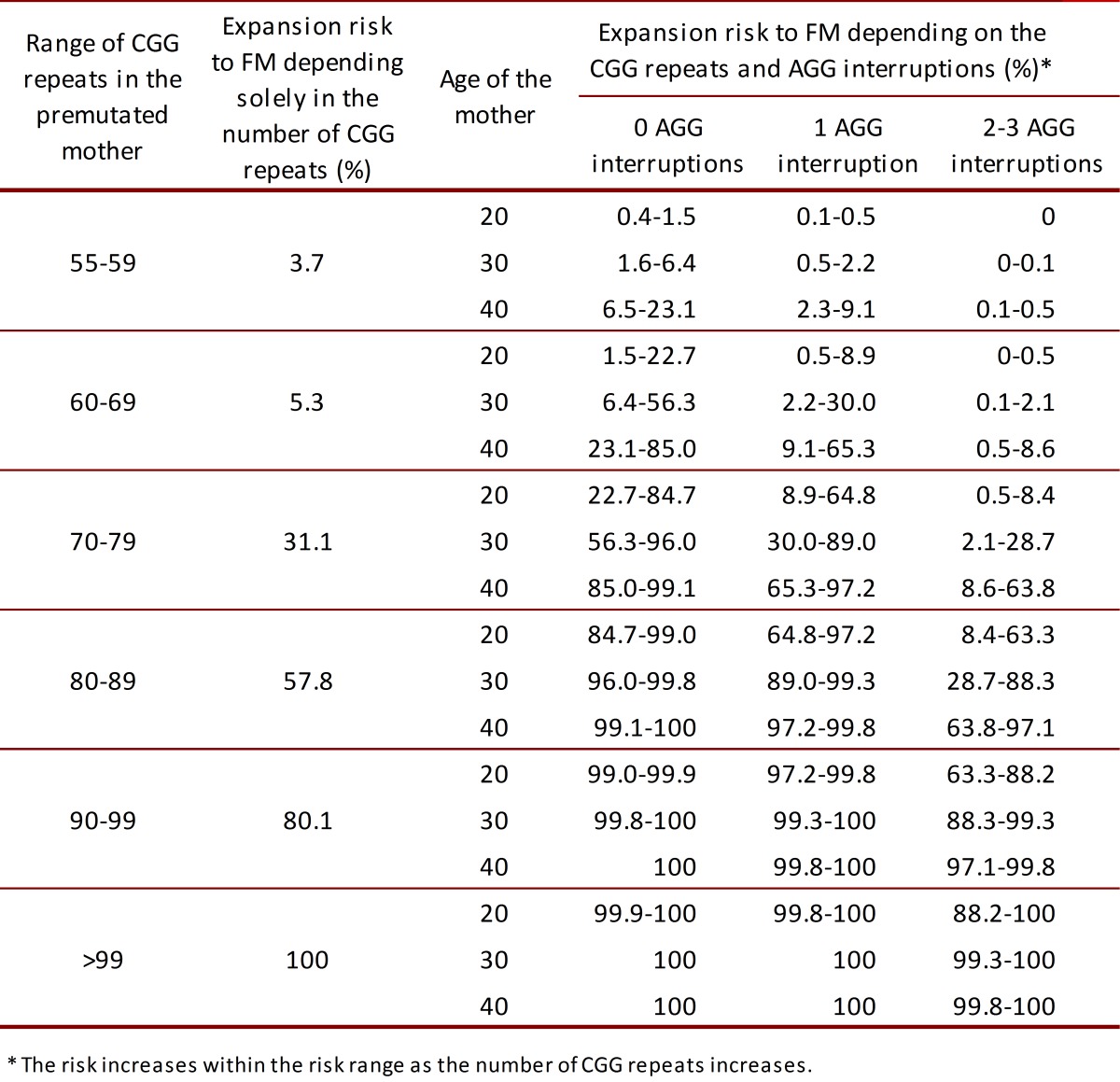
Comparison of the expansion risk from premutation to full mutation between reported percentages depending solely in the number of CGG repeats2 and recently reported percentages depending in AGG interruptions and maternal^30,31^.

The intermediate or grey zone alleles are those that possess between 45 and 54 repeats and are proposed as precursors of PM alleles. The transition from grey zone alleles to PM alleles occur because of paternal or maternal meiosis instability 32.

### 3.2. Inheritance and recurrence risk

**Men. **The majority of the men with FM usually do not reproduce with only 1% of them reported to have offspring 2. Male patients with FM and FXS have a 100% chance to pass on the premutation to their daughters so their daughters will only be carriers and usually they do not present with intellectual disabilities. There is loss of the FM in the formation of the sperm and only the premutation is passed on. All of their sons will receive the Y chromosome so they will not be affected with the *FMR1* mutation. Meanwhile, men with the PM will transmitt only the PM to their daughters and the number of repeats is relatively stable 2 ^,^ 31.

**Women. **It's noteworthy that carriers of the PM can expand their allele to FM with odds depending on the number of repeats, number of AGG interruptions and age (Table 1). The impact of the AGG interruptions is attributed to the decrease of DNA polymerase slippage in replication. Therefore, this interruptions give stability in the gene's transmission but does not affect gene's transcription nor translation 29 ^,^ 30. 

Besides, according to the newborn's sex, the clinical characteristics are going to differ. Male patients with FM will develop mental impairment. On the contrary, daughters who inherit the FM have a 30% chance to have a normal intelligence quotient, 25% to have intellectual disability with IQ less than 70, nonetheless they can present learning deficit (60%) and emotional difficulties (70%) 2.

## 4. Fragile X Syndrome clinical characteristics

The FXS cognitive and emotional phenotype will depend as well on the amount of FMRP that is produced, which depends on the number of repeats and the methylation degree of the *FMR1. *Depending on the FMRP concentration, a clinical spectrum develops. When FMRP levels are not very low the symptoms are less severe, there's a moderate emotional and learning difficulties and a normal intelligence quotient. If the production of the FMRP protein decreases or stops, a severe cognitive deficit develops causing mental impairment 2.

**Figure 1.**
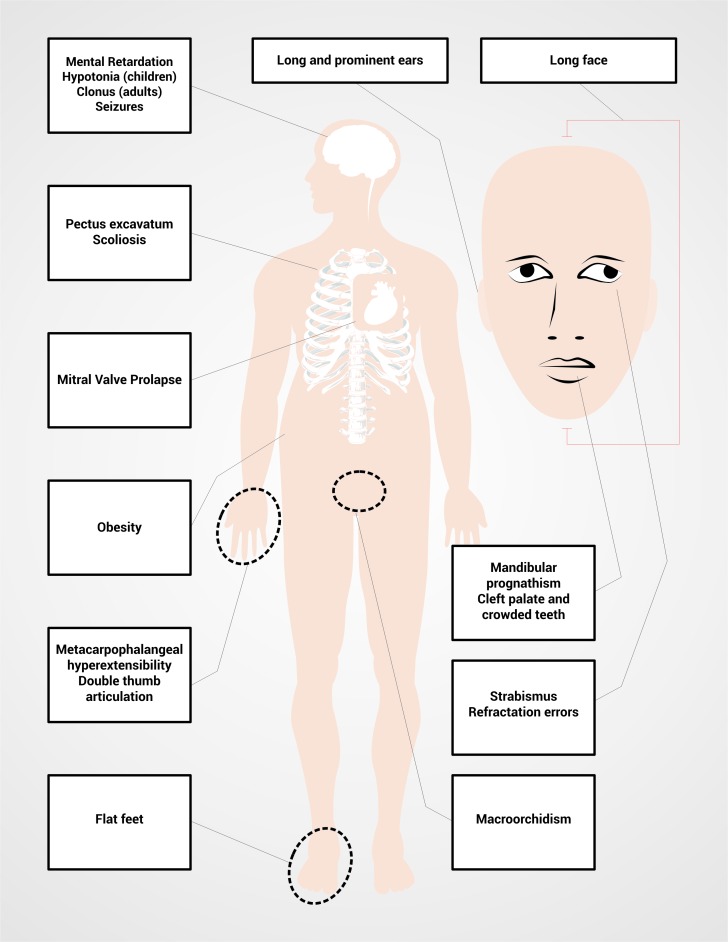
Description of the typical phenotypic characteristics of Fragile X Syndrome.

Particular physical characteristics also depend and the production of FMRP. Thus, the 80% of the patients with FXS will have one or more of their common facial characteristics (Table 2 and Fig. 1) 2. The diagnosis is suspected in men with mental impairment, particular facial characteristics as long face, large and protruded ears, and macroorchidism; this phenotype allows the distinction of patients with FXS after puberty to the patients with mental impairment and not associated with FXS 2 ^,^ 20. However all individuals with intellectual disability or autism should have the fragile X DNA test because sometimes the physical features are not obvious or not present, particularly in young children.

**Table 2.**
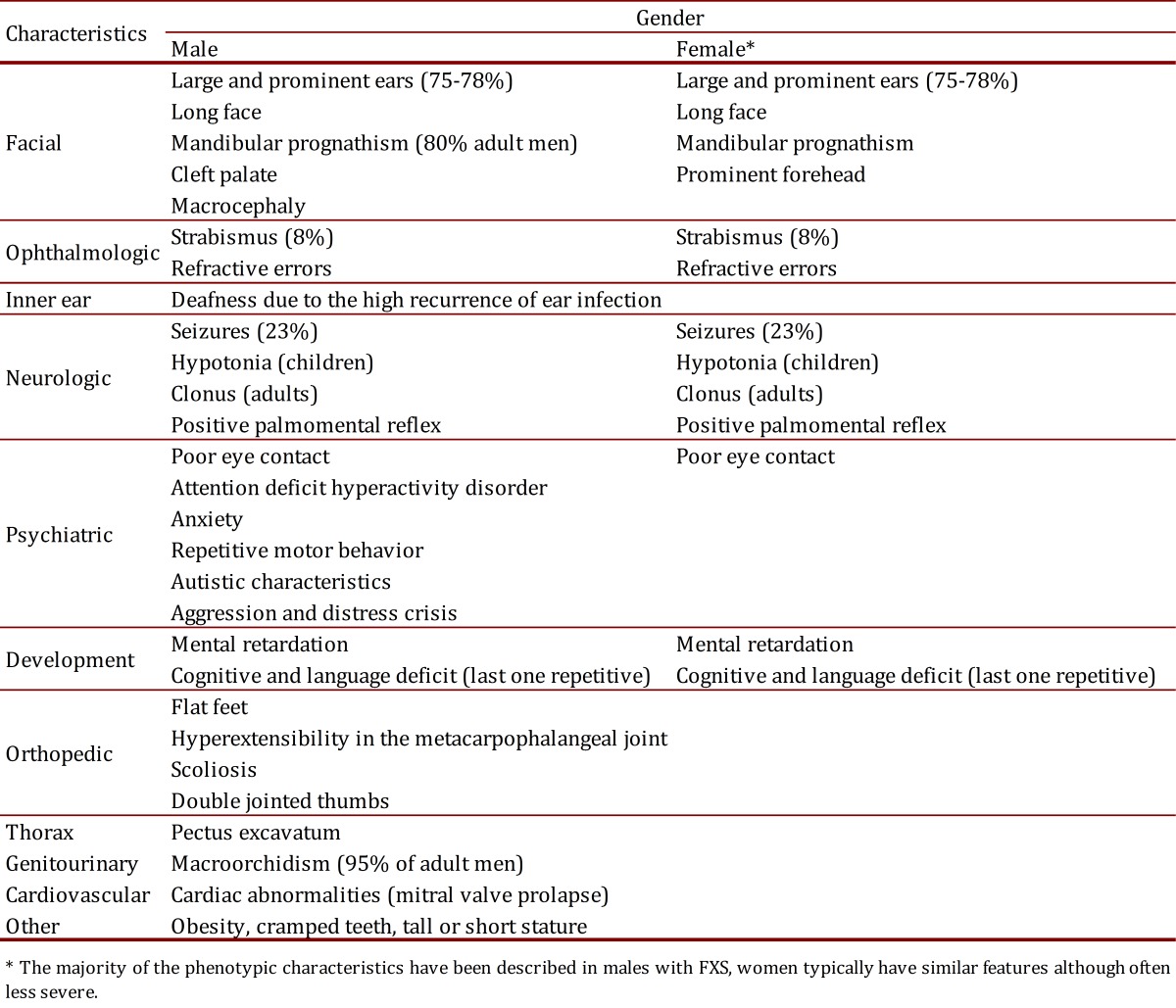
Clinical characteristics of patients with full mutation and Fragile X Syndrome^2, 21^

Women with FM and FXS are going to present a wider range of phenotypic characteristics than men, depending on the activation ratio of the affected X chromosome AR in peripheral blood (AR= percentage or ratio of cells with the normal allele present on active X chromosome, so that higher AR correspond to higher FMRP levels produced by the normal *FMR1* allele). The severity of the phenotype in women can vary from typical physical characteristics (Table 2 and Fig. 1) and mental impairment to the absence of physical phenotype together with a mild learning disability. Seventy per cent of the women with the FM present some degree of cognitive impairment 2. 

Premutation carriers usually have a normal IQ with mild or no physical features; however, approximately 20% of women develop FXPOI or FXTAS, although 40% of male carriers develop FXTAS. Tremor/Ataxia Syndrome is a late onset progressive neurodegenerative disorder 10, characterized by neurological deficits that include progressive intention tremor, cerebellar ataxia, parkinsonism, neuropathy and autonomic dysfunction 33 ^-^ 35. fragile X-associated primary ovarian insufficiency, which indicate the cessation of the menses before 40 years old, affects women by producing a series of complications in their fertility and reproduction, because it causes menstrual cycle irregularities, infertility and ovary hormonal deficiency 36. 

## 5. Diagnosis

Initially, the diagnosis of FXS was done through karyotype, which allowed the observation of the distal narrowing of the long arm of the X chromosome in the band 27.3 (Xq27.3-23.8) using the light microscope. The findings of distal constrictions can be done in different chromosomes, and are known as fragile sites, from where FXS is named 2. 

Nowadays there are several molecular tests available for the diagnosis of FXS which are far more sensitive and specific than the karyotype 11. Besides allowing the diagnosis of patients with the FM and FXS, theses tests allow the identification of carriers of the PM, which are individuals typically with a normal IQ, but the female carriers have a high risk of having children with FXS. As well there are molecular tests that allow the quantification of messenger RNA (mRNA) and of FMRP protein, allowing a better understanding of the physiopathology of the disease by correlating the results to the phenotype of the FM and PM patients 2. Polymerase chain reaction (PCR) and Southern blot are the routine tests for the DNA diagnosis of FXS which allow determining the number of CGG repeats and the methylation status of the FMR1 gene.

The PCR through the use of specific primers for the *FMR1* gene allows the amplification of the region that contains the CGG repeat and, can identify patients with an expanded *FMR1* allele particularly in the premutation but also in the full mutation range 37. Usually Southern blot analysis is utilized to better characterize alleles in the full mutation range and to determine the methylation status 38. However, with the new PCR techniques that use double primers for a nested PCR, the quantification of CGG triplet repetition and identification of FM and PM patients is possible 37 ^,^ 39.

**Figure 2.**
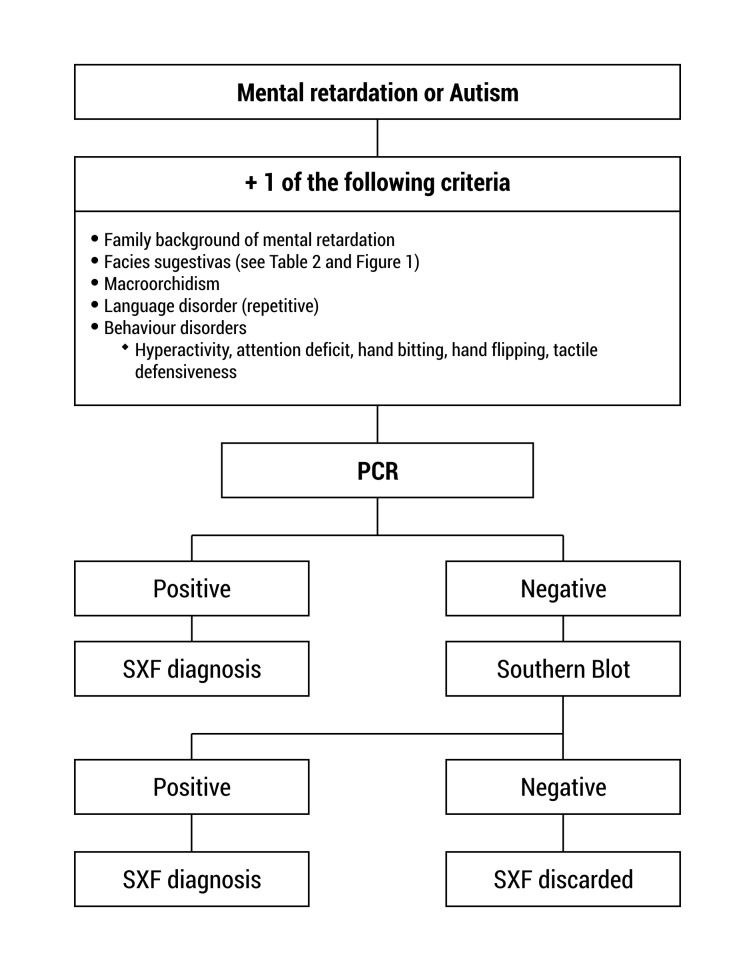
Use of molecular techniques for Fragile X Syndrome (SXF) diagnosis.

The tests for the diagnosis of FXS must be ordered for patients with intellectual disability and/ or autism and additionally any of the following features: distinct facial features as large protruded ears, long face, among others; family history of intellectual disability, autism, macroorchidism (Fig. 2)

Once it is determined that the patient has the FM or PM for FXS, DNA molecular testing must be done on all the family members that are suspected through the analysis of the pedigree of being carriers. Likewise family members with tremor, ataxia, neurological symptoms or early ovarian insufficiency would also be candidates for the molecular tests 40. 

The diagnosis of FXS can be done even in fetus of pregnant carriers or patients with FXS using the same molecular tests mentioned above (Southern blot and PCR) on chorionic villus sampling 40. 

## 6. Differential diagnosis 

The differential diagnosis includes Sotos syndrome, Prader-Willi and Klinefelter as they share particular characteristics. For each one of these syndromes there are specific molecular tests that help to confirm the diagnosis. These tests will be ordered according to the phenotypic findings and clinical analysis of the patients (Table 3) 2 ^,^ 41 ^,^ 42. The most frequent clinical findings among these syndromes that can be contrasted with FXS are: 

Sotos syndrome: intellectual disabilities, tall stature, macrocephaly, and epilepsy.

Prader-Willi syndrome: intellectual disabilities, obesity, short stature, and hypogenitalism. A subgroup of patients with FXS will have a Prader-Willi phenotype but will not have a deletion at 15 q11-13 region, although the level of CYFIP protein from this region is low.

Klinefelter syndrome: tall stature, hypogenitalism, intellectual disabilities (20%)

FRAXE: intellectual disabilities, language impairment, hyperactivity, autistic behavior (due to the abnormal repetition of CCG triplet in the FMR2 gene)

intellectual disabilities and chromosome fragility in other fragile sites have been described (FRAXD and FRAXF genes).

**Table 3.**
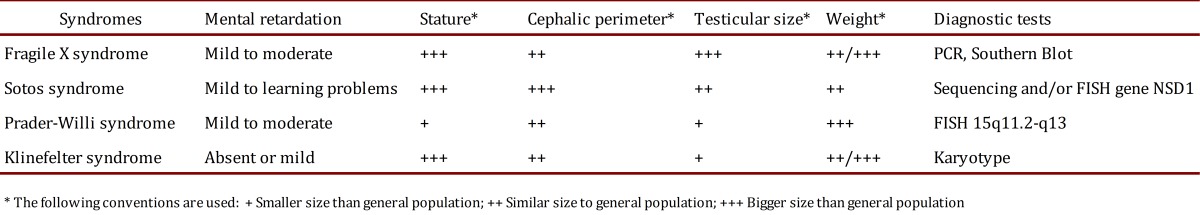
Differential diagnosis of Fragile X Syndrome^2,41,42^

Other less frequent syndromes, with prevalence lower than one in 50,000, have similar genetic and physiopathogenic mechanisms as well as phenotype to FXS. Among these syndromes there are Fragile X syndrome E and Fragile X syndrome F, due to the alteration of the *FMR2, FAM11A *and* FRAXD* genes respectively. If the phenotype of the patient highly suggests FXS and the Southern blot results come out negative, molecular testing for genes mentioned above should be considered. 

## 7. Treatment

**Figure 3.**
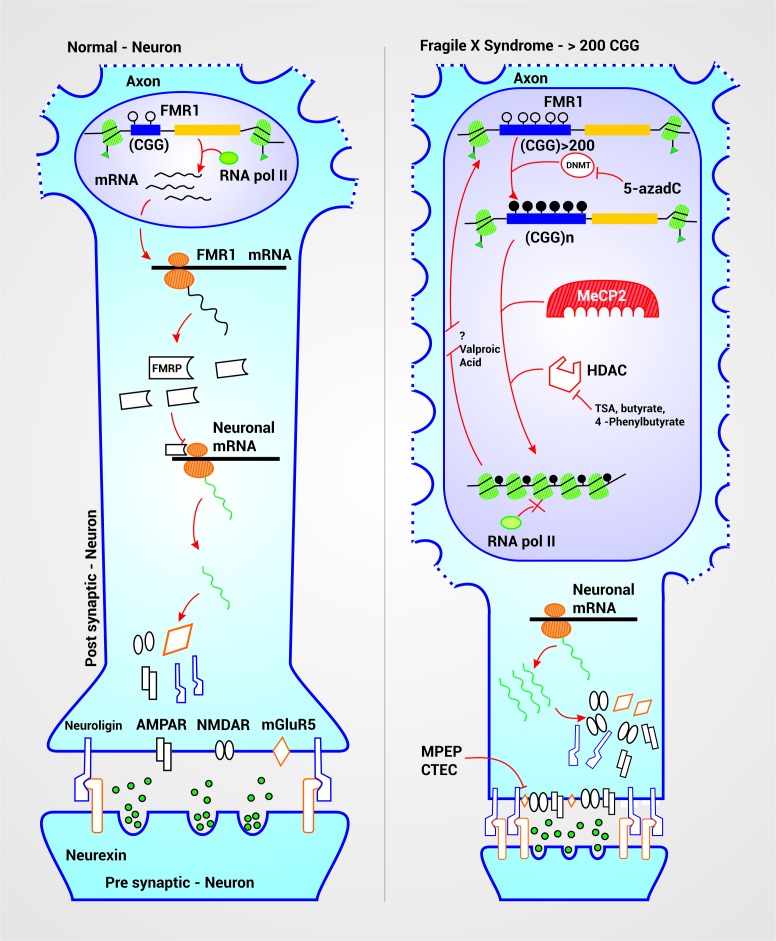
Therapeutic targets and FXS physiopathogenesis. In a neuron with a normal range of CGG repeats in the *FMR1 *gene, there is a euchromatin structural conformation which allows the entry of transcription machinery, due to the acetylation of histones H3 and H4. This conformation allows the normal production of the FMRP, protein that inhibits mRNA translation of some neuronal proteins as Neuroligin, NMDA Receptor (NMDAR), AMPA Receptor (AMPAR) and mGluR5 receptor among others. The expansion above 200 repeats leads to the detection of the CpG islands by DNA Methyl-transferases (DNMT) that add methyl groups to the cytosines of the CGG sequence and the promoter which silences the gene. This methylated cytosines are recognized by the MeCP2 which attracts and activates Histone Deacetylases (HDAC) that cleaves acetyl groups from histones H3 and H4 near the affected segment thus condensing the chromatin. Together, this changes silence the gene allowing the excessive production of neuronal proteins causing neuronal hyperexcitability, spinal dysmorphogenesis and successive clinical manifestations. 5-azadC is a drug that inhibits the DNMT preventing cytosine methylation. TSA, butyrate and 4-phenylbutyrate inhibit the HDAC therefore acting synergically with 5-azadC. Valproic acid seems to reactivate silenced genes but its mechanism has not been yet described. Inhibitors of mGluR5 allows to reduce its hyperexcitation, produced by its overproduction, and the consequences it carries.

Multiple studies have been carried out in the attempt to develop target treatments for FXS that can improve some of the symptoms and the life quality of the affected patient. These investigations have had different approaches according to their objectives, whether it is the activation of the *FMR1 *gene or the treatment of the symptoms associated to this disease. The mechanism of action of some of the medications used focus on epigenetic modulation, glutamatergic system and regulation of the translation of FMRP target mRNAs (Fig. 3.)

 Even though folic acid decreased the observation of fragile sites in the X chromosome in *in vitro* models of FXS, this drug doesn't have the same effect *in vivo*. Its benefits could derive from the role of this micronutrient in the methylation and hydroxylation of neurotransmitters, reactions implicated in their synthesis and metabolism 2. The first clinical trials with this supplement in the 1980´s reported that folic acid gave a similar positive effect to the one obtained with the use of stimulants, specifically in motor coordination, language and speech. However, the effectiveness of this drug has been difficult to demonstrate in controlled studies, limiting its potential as a possible treatment and contraindicating it for patients with seizures and FXS 2 ^,^ 43. 

Valproic acid, known as a silent gene reactivator 3 ^,^ 44, is a weak reactivator of the FM alleles in FXS 3 ^,^ 45. It has been reported that the administration of this drug to patients with FXS improves their attention deficit hyperactivity disorder (ADHD). In patients with generalized tonic-clonic seizures, absence crisis and partial seizures this is the drug of choice; as by increasing the levels of GABA and decreasing dopamine it is an excellent mood stabilizer and can be used in cases of bipolar disorder in adults and even in children 2 ^,^ 3 ^,^ 46. 

Likewise, L - Acetyl - carnitine also seems to inhibit the formation of fragile sites in the X chromosome. Its administration to patients with FXS improves significantly their attention deficit hyperactivity disorder, however it has no effect in the methylation of the *FMR1* gene, nor its expression 2 ^,^ 3. The use of this drug in the treatment of FXS patients could be indicated; however the reported studies are not conclusive.

The best medications available for treatment of ADHD symptoms in FXS are the stimulants including preparations of methylphenidate or mixed amphetamine salts. However, these medications should not be used in children under age five because they can often cause irritability 47. Nevertheless, if severe ADHD symptoms occur under age five then guanfacine or clonidine can be helpful. Clonidine can also be used to treat the sleep disturbance that is a common problem for young children with FXS. Alternatively, melatonin at a dose of 1 to 3 mg at bedtime works well for facilitating sleep in children with FXS 48.

The use of mGluR antagonists as 2-methyl-6-phenylethynyl-pyridine and CTEC, has given as a result the recovery of the dendritic spines morphology, protein synthesis, hippocampus atrophy and partially the macroorchidism in animal models 3 ^,^ 49. However, the use of mGluR5 antagonists have not demonstrated efficacy in adolescents and adults with FXS in controlled trials 2.

One of the great interests and pharmacologic targets for the treatment of FXS is the restoration of the *FMR1* gene activity by decreasing the DNA methylation and altering the H3 and H4 histones acetylation code. The use of 5-azadC (methyltransferase inhibitor) along with histone deacetylase inhibitors like TSA, butyrate and 4-phenylbutyrate have been proven to act sinergically for this purpose. Both drugs have been tested solely *in vitro*, due to the risk of inducing cellular apoptosis *in vivo* 3.

On the other hand, due the absence of FMRP protein in FXS, which regulate synaptic stimulation 50, an overproduction of metalloproteinase 9 (MMP-9) is produced in response to the normal synaptic stimulations in this disease 51, which has been associated with several pathologic conditions, particularly seizures and cerebrovascular accidents 52. Active MMP-9 regulates the pericellular environment through the cleavage of protein components. This MMP-9 function has been associated with synaptic changes associated with learning and memory 53. Treatment with minocycline for three months in patients with FXS has been proven, in some cases, to decrease MMP-9 activity independent of age and doses 54. A controlled trial of minocycline (doses 25 to 100 mg per day) in children ages 3.5 to 16 years demonstrated efficacy in behavioral improvements and with mood and anxiety problems 55. 

Minocycline can be used clinically in treatment of children and adults with FXS but when used in children under 8 it can lead to darkening of the teeth. However, when used in young children it may have its greatest effect in strengthening synaptic connections and enhancing cognitive development. The physician should discuss the side effects of minocycline with the family before prescribing Minocycline, which can also lead to loose stools usually improving with a probiotic. The darkening of the teeth can be fixed cosmetically when the child is older. Rarely minocycline can cause elevation of the antinuclear antibody and if a rash or swollen joints, visual problems or severe headache occur then minocycline should be discontinued 55.

Due to the high frequency of mood, anxiety and behavior disorders in patients with this syndrome and in carriers of the PM, serotonin selective reuptake inhibitors have been used to treat effectively aggressive behavior, anxiety, depression among others 2. The use of sertraline in FXS is a subject of current investigations; articles have reported its positive effect in language development in young children (2 to 6 years) with FXS among other virtues 56. However, in about 20% of cases an SSRI can worsen aggression because of hyperarousal especially if the dose is high. Therefore, if aggression develops with the use of an SSRI, the dose should be lowered or discontinued and a mood stabilizer, such as aripiprazole or risperidone should be started. Lithium is also an excellent mood stabilizer in FXS and a targeted treatment for FXS 47. 

Besides all of the pharmacologic options mentioned above, several authors, remark on the necessity of developing cognitive and behavior therapies, as well as educational and behavioral intervention for these patients, to be able to strengthen their social abilities, reading abilities, adaptive behavior and support network 57 ^-^ 61. Children with FXS are also typically very good with the computer so applications that focus on language, reading, math, or social deficits can be used with the help of the teacher, therapist or parent.

## 8. Conclusion

In Colombia the prevalence of FXS hasn´t been reported; however there are many patients with the full mutation and many premutation carriers with an endemic focus in Ricaurte, Valle del Cauca. The knowledge of the genomics, physiopathology of the disease and the function of the FMRP in *the central nervous system*, the heritability and phenotypical findings in the carriers have increased in the last decade. This has allowed the stimulation of multiple investigations, especially in pharmacologic interventions with targeted treatments that can reverse the neurobiology of FXS. However, the drugs that increase the FMRP levels or the expression of the *FMR1* gene haven´t been used in vivo due to safety reasons; other drugs as valproic acid, sertraline, minocycline among others, have given good results in specific symptoms of FXS patients, although they should be used according to each individual response. 
